# On Infanticide

**Published:** 1820-05

**Authors:** W. Hutchinson

**Affiliations:** Sackville-street


THE LONDON

### Medical and Physical Journal.

5 OF VOL. XLIII.] MAY, 1820. [NO. 255.

"For many fortunate discoveries in medicine, and for the detection of numerous "errors, the world is indebted to the rapid circulation of Monthly Journals; "and there never existed any work to which the Faculty in EUROPE and "AMERICA were under deeper obligations than to the Medical and Physical "Journal of London, now forming a long, but an invaluable, series." RUSH.

### Original Communications, Select Observations, etc.

### MEDICAL JURISPRUDENCE.

### DISSERTATION II.—*On Infanticide.*

### § I. *On the Physiological Relations of Infanticide.*

[In continuation from page 277.]

THE value of the several facts that have been developed in the course of this enquiry, with their relations to each other, and their application, in general, to the express object of this dissertation, having been indicated as they were passed in review, but little now remains to render complete the discussion of the present subject. A few remarks of a general character may be advanced, but these can relate only to certain points; for this is a subject on which all generalities will be found either too strict or too exclusive for many particular cases. An exposition of all those which may occur from various combinations of the circumstances already exposed, would require details that cannot be entered into in the limits of this Journal; and the medical practitioner will not often experience much difficulty in forming just conclusions, when the knowledge above developed is duly reflected on.

The appearances of the lungs, and the phenomena they display on being subjected to the tests already described, will furnish matter for positive inferences of the existence of life after birth only in a small proportion of cases; as this can only happen when they are fully dilated by air, introduced into them by *respiration*, when the pulmonary arteries and veins evince signs of their having been distended with blood, and when these organs are of a pale bright-red colour. But all these signs may exist in a greater or less degree; and it is not possible to say, in general, when they cease to present the evidence above mentioned: this however may be safely relied on,—that, when the lungs, with the heart attached to them, float near the surface of the water, in the hydrostatic test, after having been pressed in a folded cloth with all the force the hands of a man of moderate strength can exert, and when they present at the same time the signs, already indicated, of respiration, it may be considered that infantine life has been well established. When the organs just mentioned float about the middle of the water, the evidence is doubtful; and when the lungs, without the heart attached to them, float in this manner, we must find other signs of life after birth before we can permit ourselves to suppose that it has existed. The case first mentioned is but rarely witnessed when the infant has not lived several hours, and commonly, not until it has lived several days; and then there are other signs of infantine life joined with them. The first, and most easily and generally recognizable of these, are, the vital process about the umbilicus, by which the navel-string is hereafter to be separated from it; more or less complete obliteration of the cavities of the umbilical arteries, the umbilical vein, the *canalis venosus*, (that branch of the umbilical vein that passes into the vena cava,) and of the *canalis arteriosus*, (the vessel which passes from the pulmonary artery to the aorta.*) The funis separates from the umbilicus generally about the fourth or fifth day after birth, exposing an ulcerated surface, often presenting three papillous elevations corresponding to the vein and arteries; and, on cutting into it, a little yellowish watery fluid is usually found effused between the remnants of the blood-vessels, sometimes with a little redness of the surrounding portion of the peritoneum. When there is evidence of this vital separation of the funis, or of clear process towards it, joined with signs of respiration, these are proofs of the existence of life after birth.

But, infanticide is generally committed within a short time, that is to say, a few hours, and, in the greater proportion of instances, almost immediately after birth; and, therefore, before, in the generality of infants, the lungs have been fully dilated by respiration; consequently, before we can determine, from examination of the lungs and the blood-vessels, whether or not the subject has lived after its birth: but even here, the minuteness and precision inculcated in a former part of this dissertation, in the investigation of these points, will not fail to furnish highly important results. If our examination enables us to determine that *respiration* has been effected, it is often all that we desire; because infanticide is, in the greater proportion of cases, effected

* The foramen ovale in the septum of the auricles of the heart more frequently remains almost fully open for several days after birth, than becomes partially closed. The obliteration of this foramen with that also of the canalis arteriosus, is a positive sign of the existence of life after birth, *when the heart and blood-vessels are naturally formed;* but it is not such in certain cases,—for example, when the aorta springs from both ventricles.

by the commission of destructive violence, not merely by omission of proper care. There are some species of destructive violence which may not leave well-marked local signs of their having been effected during life. Respiration shows, at least, that the subject was alive after the process of parturition had commenced. When, then, we find signs of such violence as is usually resorted to in infanticide, or such as are indicative of malicious attempts to destroy the life of the subject, joined with signs of respiration, and when it appears that such violence was qualified to produce death, there are grounds for presumptive evidence of the commission of infanticide,* but not physiological proofs of it. The collateral evidence which the practitioner was directed to obtain on commencing his investigation, may prove in this case of very considerable importance. Here it is supposed, that the marks of the destructive violence do not present, in themselves, signs of their having been effected during life.

Wounds and ecchymoses, however, generally themselves present signs by which it may be known whether or not they were effected on a living subject; but careful attention is requisite in the examination of them, in order that such as often arise in the natural act of parturition may not be regarded as evidences of criminal violence. Very extensive injury is sometimes effected on the head by the use of forceps, and other instruments employed in the artificial delivery of the fetus; and dislocations and fractures of the limbs sometimes happen from the operation of turning it in the uterus: it will therefore be of importance that the enquirer should be acquainted with the circumstances attending the delivery, when professional assistance has been exerted.† The species of injury which commonly arise from the natural act of parturition, have been described in a former part of this dissertation.

When an infant has perished from want of proper care, there is generally great difficulty in determining whether or not it had lived after its birth; and this cannot be done after the most assiduous investigation in a great proportion of cases. It has been shown, that respiration is but very imperfectly effected when the usual fostering management of infants is not employed; and they frequently die, in this case, before any of the

---

* Perhaps, properly speaking, the human progeny may not be termed an infant until after its birth; but, in the present subject of jurisprudence, a fetus is regarded as an infant in its passage from the uterus during natural parturition: it is considered that the murder of a fetus under such circumstances is *infanticide*.

† One species of destructive violence, that of *poisoning*, has not been noticed in this dissertation; this will be treated of in the section relating to *homicide*.

remarkable appearances about the navel or in the blood-vessels peculiar to the fetus, designated on a former occasion, can be observed.

If a newly-born living infant be left in a certain place, it may happen that the feces may be found evacuated from it; and, if the placenta be separated, and the navel-string divided near the abdomen, there may be signs of hemorrhage having taken place from the infantine extremity of the funis, with the blood collected on the spot; both of which circumstances will show it to have lived after being placed in the situation in which it has been found: only care must be taken to ascertain that the blood found on the spot has come from the infant, by examination of its internal organs; because the blood may be that of the mother. Moreover, though it appear that an infant has perished from hemorrhage, it cannot be concluded that this has taken place from the navel-string, except it be proved that it could not have happened from any other part of the body. The great importance of very accurate examination of all the natural canals and cavities of the body, as well as of its external surface, is here very forcibly shown. Here some facts present themselves, which add to the difficulties already noticed that envelop this subject. An infant may be born in a state of such torpor, that it is apparently dead, and yet life may afterwards become well established. Physiology will indeed show us that the circulation may proceed in a newly-born infant without respiration, provided the foramen in the septum of the auricles of the heart, and the *canalis arteriosus*, remain pervious; and it is rendered evident by facts, that the change of the blood from venous to arterial, effected by respiration, is not essential for the continuation of the phenomena of life for a certain length of time in the newly-born infant. Bohn relates* three cases which he himself witnessed, that are conclusive on this point: these were cases where infants had been deeply buried in the earth almost instantly after their birth, and taken thence living several hours afterwards. Corollary to this point is the fact, that infants, at the full term, have been expelled from the vagina enveloped in the entire membranes, and lived and moved for some time in this state.†

These, and many other analogous facts that might be ad-

* *Tract. de Offic. Med. For.* p. 667.

† The most clearly-marked instances of this on record that occur to my recollection, are two related by Wrisberg. In one instance, the infant lived seven minutes thus enclosed in its membranes; in the other, nine minutes. Separating the external membrane, he observed them through the internal one. They were curved forwards, and immersed to half the depth of the body in the fluid of the amnios; moved their arms and legs, the former in a direction from the face to the chest; the latter were alternately a little elongated and retracted. The mouth duced, prove that an infant may live after its birth without breathing: in this case it might be eventually destroyed by putting it under water, or by obstructing the passages of the mouth and nostrils by any means; and we could not assert that it had lived after its birth. Here our want of knowledge may be the means of saving the life of a criminal: this may excite feelings of regret; but these are incomparably less distressing than those which must arise in the mind of every intelligent physiologist, who contemplates the records of criminal justice in the different countries of Europe for a few centuries past, and sees on what grounds capital punishments have often been attached.

It is not necessary for me to dwell again on the means by which the life of a newly-born infant may be destroyed: those peculiar to this stage of life have been already designated; the rest are common to human life in general. This may, however, be now stated, that, unless seven months of fetal life have been exceeded, the probable inference is, that, although respiration may be established after birth, the subject will soon cease to live, notwithstanding every proper care. The cases in which infants have been preserved alive for a considerable time, that had not arrived beyond the term of six months and two weeks of fetal life, are very rare. This fact, too, should never be lost sight of, that a great proportion of infants die soon after their birth, in spite of all the attentions that natural instinct and medical precepts inculcate as best calculated to preserve their life.

Supposing it to be apparent that an infant has lived for some time after its birth, and that it has died from want of proper care, the question arises, whether or not the mother (supposing her to have been delivered whilst alone) was able to exert this care. In order to decide this, it will generally be necessary to take into consideration several *moral* as well as physical circumstances: those which it is the duty of the medical practitioner to investigate were designated at the commencement of this dissertation; but, as it is on moral circumstances that the decision of the question must often be founded, it passes then from the medical practitioner to the court of judicature.

The care and succours which newly-born infants require, and without which they will generally die, are the following: 1°, To remove them from the state of supination in which they generally lie on their expulsion from the vagina of the mother; 2°, to preserve about them a degree of heat nearly equal to that of the medium they have just quitted; 3°, to supply them with

---

was closed, and no movement of the thorax or abdomen could be discerned. Fearing that the gratification of his curiosity any longer might be criminal, he now ruptured the membranes.

proper aliment; 4°, to divide the navel-string, and apply a ligature to it.

Infants are generally born with the face turned towards the sacrum of the mother, and remain lying on their belly, if the woman has been delivered in the horizontal position. Here, HUNTER remarks, " a strong child may be born perfectly alive, and die in a very few minutes for want of breath; either by being upon its face in a pool made by the natural discharges, or upon wet clothes; or by the wet things over it collapsing and excluding air, or drawn close to its mouth and nose by the suction of breathing." This fact he illustrates by the following case: " A lady, at a pretty distant quarter of the town, was taken with labour-pains in the night-time. Her nurse, who slept in the house, and her servants, were called up, and I was sent for. Her labour proved hasty, and the child was born before my arrival. The child cried instantly, and she felt it moving strongly. Expecting every moment to see me come into her bed-chamber, and being afraid that the child might be someway injured, if an unskilful person should take upon her the office of a midwife on the occasion, she would not permit the nurse to touch the child, but kept herself in a very fatiguing posture, that the child might not be pressed upon or smothered. I found it lying on its face, in a pool which was made by the discharges; and so completely dead, that all my endeavours to rouse it to life proved vain." This fact is of much importance on this occasion, but the case is one that is not likely to happen often: it is for the jury to determine whether the conduct of the mother, in a similar one, has arisen from malice or from erroneous prejudice. An infant may lose its life in the same way by the state of the mother, when delivered alone, rendering her unable to remove it from such a situation. The most common of such states are, mental alienation, a certain degree of faintness or complete syncope, various states of coma or stupor, apoplexy, and epilepsy: instances of which occurring at the time of the expulsion of the fetus, are related by SMELLIE, DE HAEN, ROOS, CHRISTIE, and almost every writer on the obstetric art, whose observations are drawn from extensive experience. Such states as those just mentioned, or the prejudices of the woman whose case is related by HUNTER, are the only circumstances which can excuse the leaving the infant in the situation in question, because the natural instinct of the mother leads her to take up her child, and to foster it with proper care. The medical practitioner may in some cases be able to derive some degree of presumptive evidence of the occurrence of such states, from subsequent examination of the mother; and he may thus furnish the jury with more or less solid grounds for the establishment of their verdict, in cases where such a plea is set up by the delinquent; but this is a point which much more frequently rests on the basis of moral evidence alone, as existing in various circumstances connected with the conduct of the delinquent before, and subsequently to, her delivery. This should, however, be impressed on the minds of the jury,—that it is possible for a woman to be with child without her supposing that she is in this state.* The circumstance of an unmarried woman undergoing labour alone, in the midst of civilized society, and of not having prepared linen, &c. for her infant, are generally regarded as unfavourable to her. It is for the medical practitioner to state, that, supposing her to be conscious of her pregnancy, she may have miscalculated the epoch of her delivery: the other moral circumstances of the case appertain to the jury. Estimable authors on the obstetric art have stated, that an infant may die from suffocation, if it lie on its back for some time after its delivery, apparently from the glairy mucus collected about the fauces getting into the trachea. The remarks made on the former case are equally applicable to the latter.

The succours mentioned in the second and third propositions (page] 357) are so constantly afforded when the mother is directed by her natural instinct, that the want of them must be considered to arise either from criminal neglect, or from some of the states of bodily ailment in the mother, mentioned above, rendering her unable to supply them.

The proposition respecting the division of the navel-string, and the application of a ligature to the infantine portion of it, involves three or four difficulties; which consist in the following cases. But, let me first remark, that the instinct of a woman leads her, when she hears her infant cry, or finds it is delivered, to take it up, and tear asunder the navel-string with both her hands; by this means it must be torn at more than a hand's breadth from the umbilicus: in this case hemorrhage from the infant will hardly ever take place. The cases involving the principal difficulties are these: 1°, The infant may perish during its birth, from hemorrhage from the placenta or rupture of the navel-string, and the mother may or may not have divided the latter; 2°, the child may have lived after its birth, and the mother may have torn or cut asunder the navel-string, and, finding no hemorrhage ensue, she has not been led to put a ligature on the infantine portion, and afterwards hemorrhage has

* Besides the common ignorance of young girls on this subject, it may be worthy of remark, that there is very prevalent amongst women the notion that the occurrence of any thing like a *menstrual evacuation* shows, certainly, that they are not with child. Nothing is better proved, than that such a discharge does appear in some women throughout the whole of their pregnancy. The source of such a discharge is not a subject for discussion in this place.

taken place from it, from which the infant has died;* 3°, the mother may discover the hemorrhage in the last-mentioned case, and may apply a ligature to the navel-string, but too late to preserve the infant's life; 4°, the blood of the mother may be artfully placed about the child, and the navel-string left untied, and the mother may wish to have it appear that the infant perished from hemorrhage occurring unknown to her, and that she was not aware of the necessity of tying the navel-string, even though it be found that she has *cut* it, not *torn it asunder* with her hands.† In the first three cases, there will be present the signs of such hemorrhage, as described in a former part of this dissertation, (page 184): in the last case, the proper fulness of the blood-vessels and heart with blood will show the imposture. It is impossible to trace any rules of general application respecting the first three cases. Our decision must be partially founded on various collateral physical and moral circumstances, which have been already designated in this dissertation and discussed in regard to their value and various relations to this subject. There will, certainly, be much difficulty in forming a correct decision, and sometimes impossibility to do this, in some cases of the kind just indicated, even after the most patient and accurate investigation of all the circumstances we can collect respecting them; but, fortunately for justice and humanity, instances of death of infants from these causes are not of frequent occurrence.

In the case of infants being found in *cloacas*, and such like places, it should be understood that an infant, even at the full term of utero-gestation, may escape from a woman who has borne one or more children, during her exertions to evacuate the contents of the intestines; and this may even happen without her being conscious of it. Cases of the fact itself are related

---

* The following case lately occurred to a medical practitioner of my acquaintance: The navel-string of a living infant was tied in the usual way, but by accident the funis was separated very close to the ligature. Two hours afterwards he was sent for; and, on his arrival, he found the infant on the point of dying from hemorrhage, that had just occurred from the navel-string. The infant had been washed and dressed in the usual way, and had not cried after it had been placed in bed with the mother, soon after which the hemorrhage was discovered. It should, however, be borne in mind, that hemorrhage of this kind is very unlikely to occur when the navel-string is torn asunder with the hands. [*See the ensuing note.*]

† The division of the navel-string by an instrument may properly give rise to a strong presumption that the mother is acquainted with the practice of civilized persons in this point, and therefore that she knew that a ligature should be applied to it: besides, the division of the navel-string in this way is almost always instantly followed by some degree of hemorrhage from the infantine portion of it; in which case there can be no doubt but that the mother's instinct would lead her to grasp it, and look about for something to tie it with, in order to staunch her infant's flowing blood.

by estimable authors, on grounds which cannot be disputed: and an instance* in which it occurred unknown to the mother, equally valid, has been published by Klein.†

*Sackville-street; March 4th*, 1820. W. HUTCHINSON.

---

FOR THE LONDON MEDICAL AND PHYSICAL JOURNAL.

### *Researches respecting Animal Heat.* By James Down, Esq.

* In the *Jahrb. der Deutschen Med.* b. iii. heft. i. p. 48.

† Here terminates that part of the present dissertation which relates expressly to infanticide in its physiological relations; but one point corollary to it yet remains to be considered,—which is, the determining, as nearly as possible, from the appearance of the body of the infant, the period that has elapsed since its birth. This is a matter of importance, when we are required to determine whether it is probable that a woman, supposed to be the mother of an infant that may have been found in a retired place, does really bear the relation to it above indicated. The discussion of this point, with the laws relating to this subject, will be given in the next Number.

